# Using Bioconductor Package BiGGR for Metabolic Flux Estimation Based on Gene Expression Changes in Brain

**DOI:** 10.1371/journal.pone.0119016

**Published:** 2015-03-25

**Authors:** Anand K. Gavai, Farahaniza Supandi, Hannes Hettling, Paul Murrell, Jack A. M. Leunissen, Johannes H. G. M. van Beek

**Affiliations:** 1 Centre for Integrative Bioinformatics, VU University, Amsterdam, The Netherlands; 2 Netherlands Consortium for Systems Biology (NCSB), Amsterdam, The Netherlands; 3 Section Functional Genomics, Dept. Clinical Genetics, VU University Medical Center,Amsterdam, The Netherlands; 4 Department of Statistics, The University of Auckland, Auckland, New Zealand; 5 Laboratory of Bioinformatics, Wageningen University, Wageningen, The Netherlands; 6 Institute of Biological Sciences, Faculty of Science, University of Malaya, Kuala Lumpur, Malaysia; Mayo Clinic, UNITED STATES

## Abstract

Predicting the distribution of metabolic fluxes in biochemical networks is of major interest in systems biology. Several databases provide metabolic reconstructions for different organisms. Software to analyze flux distributions exists, among others for the proprietary MATLAB environment. Given the large user community for the R computing environment, a simple implementation of flux analysis in R appears desirable and will facilitate easy interaction with computational tools to handle gene expression data. We extended the R software package BiGGR, an implementation of metabolic flux analysis in R. BiGGR makes use of public metabolic reconstruction databases, and contains the BiGG database and the reconstruction of human metabolism Recon2 as Systems Biology Markup Language (SBML) objects. Models can be assembled by querying the databases for pathways, genes or reactions of interest. Fluxes can then be estimated by maximization or minimization of an objective function using linear inverse modeling algorithms. Furthermore, BiGGR provides functionality to quantify the uncertainty in flux estimates by sampling the constrained multidimensional flux space. As a result, ensembles of possible flux configurations are constructed that agree with measured data within precision limits. BiGGR also features automatic visualization of selected parts of metabolic networks using hypergraphs, with hyperedge widths proportional to estimated flux values. BiGGR supports import and export of models encoded in SBML and is therefore interoperable with different modeling and analysis tools. As an application example, we calculated the flux distribution in healthy human brain using a model of central carbon metabolism. We introduce a new algorithm termed Least-squares with equalities and inequalities Flux Balance Analysis (Lsei-FBA) to predict flux changes from gene expression changes, for instance during disease. Our estimates of brain metabolic flux pattern with Lsei-FBA for Alzheimer’s disease agree with independent measurements of cerebral metabolism in patients. This second version of BiGGR is available from Bioconductor.

## Introduction

Metabolism directly reflects cellular functioning. If the biochemical reactions that operate in a cell type are known together with uptake or release measurements of some metabolites, the distribution of metabolic flux in the metabolic system can often be predicted. Large scale reconstructions of metabolic networks are valuable resources for building models for flux estimation. In recent years, genome-scale metabolic networks have been reconstructed for various organisms, such as microorganisms, animals and humans [1]. Public resources, for instance the BioModels database [2] and the BiGG database [3], exist that store metabolic reconstructions in the standard modeling format SBML [4]. BiGG stores reconstructions of metabolism consisting of large lists of metabolites and reactions for *H*. *sapiens*, *M*. *barkeri*, *S*. *cerevisiae*, *H*. *pylori*, *E*. *coli*, *P*.*putida*, *M*. *tuberculosis* and *S*. *aureus*. The new version of the metabolic reconstruction for *H*. *sapiens*, Recon 2 [5], is presently the most comprehensive reconstruction of human metabolism. The reconstructions recorded in these databases consist of genes, proteins, metabolites and reactions that are connected with each other, forming metabolic networks.

Constraint-based modeling has proven to be effective to analyze the functional states of metabolic networks when the structure of the network is known (see e.g. [6]). The parameters to be estimated are the feasible reaction fluxes throughout the network. The reconstruction databases provide the stoichiometry of a large number of reactions in many metabolic systems. Constraints are imposed to narrow the solution space. Often an optimum value is determined of a certain function of the network, for instance maximal ATP synthesis or maximal biomass production.

Several FBA software tools support import from databases [7]. The free COBRA 2.0 toolbox [8] accesses the reconstructions in the BiGG database, but requires the commercial software package MATLAB. The first version of the open source package BiGGR was to our best knowledge the first R package for constraint-based modeling of metabolic systems and has been available for several years from the CRAN repository [9]. An initial version of BiGGR was described in [10]. Since then two other R packages appeared for constraint-based modeling. The sybil package [11] is an object-oriented implementation of constraint-based modeling methods in R with emphasis on fast computation of large problems. The abcdeFBA package [12] has a more generic approach, but does not appear to include ensemble modeling and network visualization. Recently, an open source object-oriented implementation of constraint-based modeling in Python, COBRApy [13], appeared.

Here we demonstrate the substantially extended second version of BiGGR which is specifically designed for automated model generation and visualization of FBA results and was recently included in the Bioconductor open source project for bioinformatics. Emphasis is placed on functionality to assess the uncertainty of flux distributions estimated from measurements of metabolite uptake and release from the cells under study. This is accomplished by sampling ensembles of flux distributions using Markov chain Monte Carlo (MCMC) approaches. We further demonstrate incorporation of gene expression changes in metabolic analyses. BiGGR is still a relatively lightweight, low-threshold implementation of constraint-based analysis with direct access to reconstruction databases to import reactions. It links models built from the metabolic reconstructions with linear inverse modeling routines developed by ecologists [14] which turn out to be very convenient to analyze metabolic models. Many analyses for genomics, metabolomics and proteomics studies require extensive use of statistical libraries that are readily available within the R [15] and Bioconductor [16] frameworks. These libraries can be combined together to form an integrated workflow for analysis and interpretation of “omics” data. BiGGR provides an additional feature to construct models based on simulation as well as visualize these results using the “hypergraph” [17] framework. This framework is an intuitive and appropriate way to visualize chemical reaction networks. This representation is helpful to an investigator to analyze results and examine model results for further analysis.

We propose that BiGGR is a relatively easily accessible way to analyze metabolic networks, especially for users of R and Bioconductor. Tools available in R and Bioconductor for handling ‘omics’ data can readily be combined with BiGGR. Below we therefore demonstrate the functionality of BiGGR by estimating metabolic fluxes in brain from measurements of metabolite exchange and gene expression in healthy humans and patients with Alzheimer’s disease. To this end we developed and demonstrate a new algorithm, termed Least-squares with equalities and inequalities Flux Balance Analysis (Lsei-FBA), which predicts changes in metabolic flux distribution from changes in gene expression between health and disease.

## Methods

### Software Features

The R package BiGGR comprises the databases from BiGG [3] and the recent reconstruction of human metabolism Recon 2 [5] as SBML objects in R. Other metabolic reconstructions can be imported, e.g. from the BioModels database [2]. BiGGR provides functionality to query the databases for specific pathways, reactions or genes and select the entire network or sub-networks to which FBA can be applied. FBA is conducted by converting a network into a linear inverse model, which is then solved using linear programming algorithms [14,18]. In addition to model assembly and analysis, automatic visualization of selected parts of metabolic reconstructions with estimated reaction rates is implemented using hypergraphs which provide graphically intuitive plots of biochemical pathways [17]. BiGGR is available at http://bioconductor.org/packages/release/bioc/html/BiGGR.html. A detailed tutorial is available as a vignette within the package documentation.

**Fig 1:**
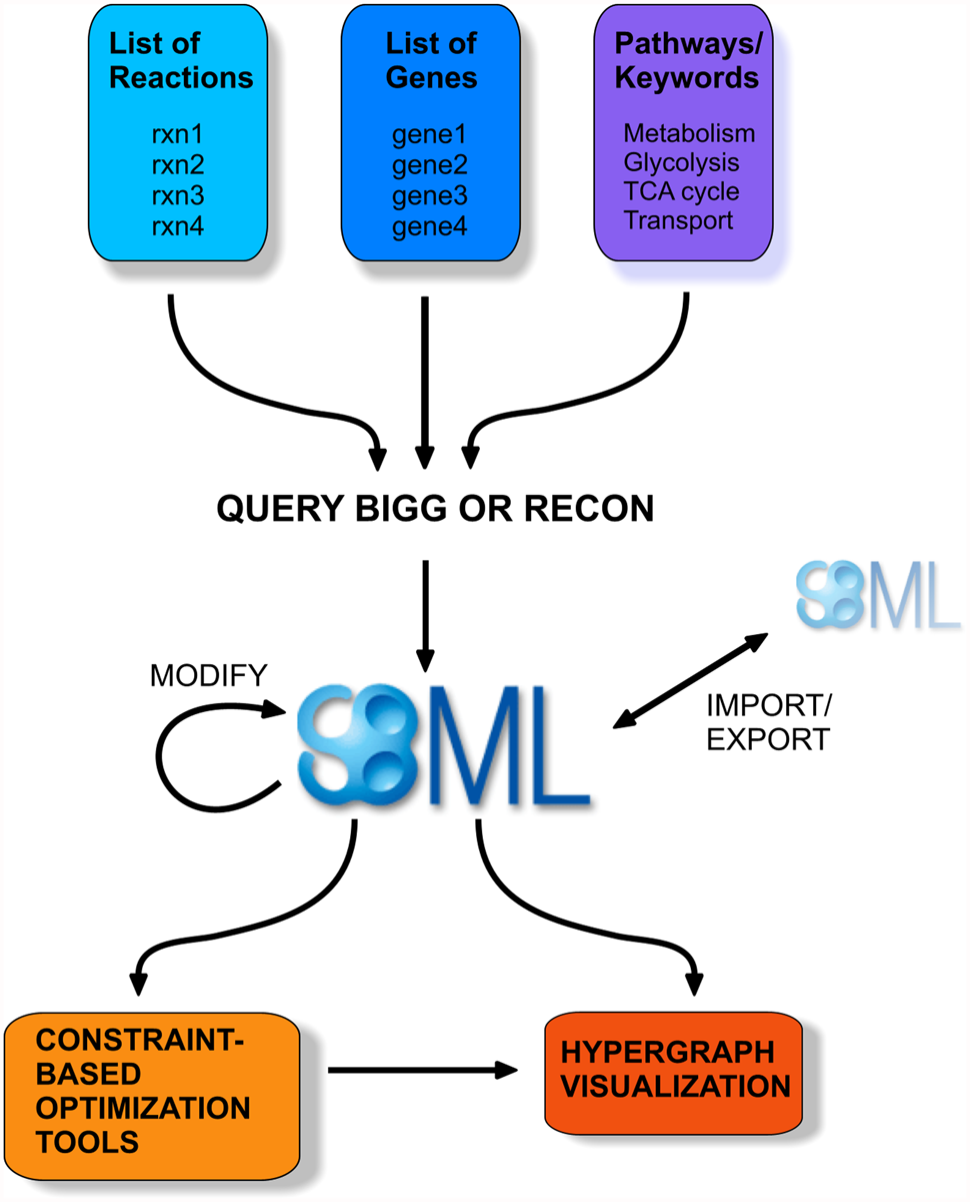
Overview of BiGGR functionality.

BiGGR provides the following functionalities (see Fig. 1 for a graphical summary):

### Overview of the functionality of the BiGGR package

#### Model creation

Models can be assembled by querying the metabolic reconstruction databases for specific pathways (e.g. glycolysis, TCA cycle). All reactions with annotations indicating that they belong to a certain pathway can be imported in one go. Further, reactions can be imported by specification of lists of reactions, metabolites or gene identifiers. Irrelevant reactions can also be removed.

#### Model import/export

Models in SBML format can be easily imported into BiGGR for further analysis. Model files exported from the web interface of the BiGG database (bigg.ucsd.edu) can also be imported. Each model created or modified within BiGGR can be exported in SBML format.

#### Flux estimation

BiGGR uses linear inverse model (LIM) approaches for flux estimation. The fluxes in an underdetermined system can be calculated based on a linear function (i.e. a weighted sum of the unknown variables) which is either minimized (a “cost” function) or maximized (a “profit” function). The function to be minimized or maximized can be subject to inequality constraints, e.g. due to irreversibility of reactions which cannot carry negative fluxes, and equality constraints which reflect the metabolic steady state assumption. BiGGR can also generate ensembles of parameter combinations with a probability density reflecting the likelihood of explaining measured data.

#### Visualization

BiGGR provides automatic visualization of a network with hypergraphs using the hyperdraw package [19]. The graph displays metabolites connected with each other using hyperedges which represent reactions. Edge widths represent the intensity of estimated fluxes. It usually works best to plot a selected subset of metabolites or reactions.

An overview of flux balance and ensemble modeling methods implemented in BiGGR is given below. This is followed by a description of the methods used to predict redistribution of fluxes based on measurement of changes in gene expression during disease. In the results, examples of the application of these methods are given.

### Flux Balance Analysis in BiGGR

Flux Balance Analysis (FBA) is a constraint-based modeling method which derives the vector of fluxes by optimizing an objective function while subject to given constraints. The objective function can either be a *profit* or a *cost* function for maximizing or minimizing certain (combinations of) fluxes given the constraints. Constraints can be equality constraints, such as the steady state assumption and inequality constraints, e.g. posed by the irreversibility of reactions. FBA is a widely used modeling technique and therefore not explained in detail here. For more information, the reader is referred to a large body of literature on FBA, e.g. [6].

In brief, FBA uses a linear programming routine to optimize a flux vector *x* given a cost or profit function with respect to the constraints:

Ex = f (1)

Gx ≥ h (2)

Matrix *E* is the stoichiometry matrix of the given reaction system with the rows representing internal metabolites that should be balanced to zero, but without rows that represent external metabolites which are not balanced to zero. These external metabolites may for instance accumulate or decrease in level in the medium outside the cells in a cell culture, or may be metabolites in the cell which are used in unknown reactions not yet fully accounted for in the model. The entries in the vector f are usually set to zero. The first equation therefore ensures that the optimized flux vector *x* is in the null space of the stoichiometry matrix and therefore the assumption of metabolic steady state holds. In some cases in an FBA, additional equality constraints are desirable, if for instance the value of a certain flux is known beforehand with great precision. The flux can then be fixed to this reference value using matrix *E* and vector *f*.

The inequality constraint *Gx ≥ h* usually constrains fluxes in irreversible reactions. Matrix *G* therefore indicates irreversible fluxes and the vector h consists of zeroes. In BiGGR, FBA is conveniently performed by interfacing the model with the linear programming routines in the ‘LIM’ package [14]. Equality constraints for fluxes that were determined experimentally can be easily set. If a model is assembled from a metabolic reconstruction database, BiGGR automatically sets reversibility constraints for reactions that are defined as irreversible in the database.

The constraints represented by *E* and *G* matrix can be combined with a linear function that specifies (a combination of) fluxes to be minimized or maximized. Maximization of ATP production (see equation 4 in Results section) is an example of such a linear programming approach.

### Sampling of feasible flux distributions

If the measurement error of experimentally determined fluxes is known, it would be of interest to know how the estimates of the rest of the fluxes are sensitive to this error. In order to predict the sensitivity of model estimates with respect to uncertainty in the values of model parameters, MCMC methods can be used to sample ensembles of parameter sets that agree with experimental data within a certain error [20,21]. By sampling an ensemble of flux parameters, the uncertainty in all estimated fluxes is quantified directly. Measured fluxes are allowed to vary in the ensemble within their measurement errors. It is important to note that for all flux vectors in the ensemble, the equality and inequality constraints (see above) still hold and equations 1 and 2 still apply. The sampling procedure must therefore sample within the feasible region of the solution space which is spanned by the given constraints. With the approximate equality constraints added via matrix *A*, an ensemble of feasible flux vectors is generated where the density of points in the ensemble is proportional to the likelihood of that region in the parameter space explaining the measured data.

Ax = b + ϵ (3)

*A* is the matrix containing the information of which fluxes were measured, *b* contains the measured flux values and ϵ is the measurement error. The sampling procedure then constructs a series of samples *x* for which *Ax—b = ϵ* To this end, BiGGR uses the *xsample* function from the LIM package [18] which in turn uses the Metropolis-Hastings algorithm to sample a posterior distribution of flux vectors within the given constraints. The measured values of fluxes, for instance exchange fluxes with the culture medium in a cell culture or between blood and brain (see Results), are entered in b while the measurement error for ϵ is given by the standard error of the measurements. The MCMC algorithm produces an ensemble which approximates a probability density function giving the likelihood of a point in flux parameter space given the measured exchange flux data. In BiGGR, approximate equality constraints and measurement errors can be entered in a convenient fashion.

To sample a posterior distribution of feasible flux vectors, we used the BiGGR function *sampleFluxEnsemble* which provides an interface to the *xsample* function in the LIM package. Metabolic exchange rates for glucose, lactate and pyruvate and their standard deviations (values are given in the ‘Results section’) were passed to the sampling function. Sampling was performed with the ‘mirror’ algorithm implemented in *xsample*. After an initial burn-in of 10^7^ Monte-Carlo steps, we sampled 10^7^ flux vectors, from which every 100^th^ vector was included in the ensemble, resulting in an ensemble of size 100000. While neither burn-in or thinning of the Markov chain to minimize autocorrelation are mandatory practices [22], they reduce the amount of data saved from an MCMC run, which makes further analyses and visualization convenient. Convergence of the algorithm was assessed by a visual inspection of the trace for each estimated flux in the ensemble and by calculating the autocorrelation function. The ‘jmp’ parameter determining the relative proposal step size of the Metropolis-Hastings algorithm was adjusted to 0.1 to ensure convergence.

### Analysis of flux distribution based on gene expression changes

In the example presented below, the aim was to predict changes in metabolism based on measured changes in gene expression data of Alzheimer’s disease (AD) patients (see Results). Microarray CEL files were downloaded from the Gene Expression Omnibus with the accession ID GSE5281 [23]. This dataset contains microarray gene expression measurements from laser captured micro dissected neurons from healthy and AD subjects. For the present analysis, only the hippocampal region, which is the region most affected during the early stages of the disease is used. The CEL files were preprocessed and normalized using the RMA method from the R package *limma* [24]. Log2 fold change values were used to calculate differences in expression levels of the AD patients against healthy controls. Probeset to gene annotation is extracted from the Affymetrix annotation library in Bioconductor using the package *annotate*. Subsequently, annotations linking genes to reactions were extracted from the Recon1 database in the BiGGR package using the *extractGeneAssociations* function. In case of multiple genes linked with a reaction, the average of the fold changes is computed.

In the following part of the Methods section we detail the computational procedure to calculate changes in metabolic fluxes from changes in gene expression. The rationale for this computational approach is discussed further in the Results. We started from the flux distribution in normal healthy brain, determined using FBA with total ATP production maximized and with the pentose phosphate pathway (PPP) and GABA shunt fluxes constrained to measured values relative to glucose uptake. For the diseased brain, an initial estimate of the fluxes in the AD patients is first computed by multiplying the flux estimate in each reaction in normal brain with the fold change in gene expression for the genes mapped to that reaction. In the second step, this initial rough flux estimate was refined by taking the flux balance (equality) and irreversibility (inequality) constraints into account, see equations 1 and 2. To compute the final prediction a cost function was minimized by putting the initial rough estimate in the *b* vector of the *Ax = b* equation and providing a reference in the *A* matrix to the corresponding flux. In this way the sum of the squares of the differences between the final estimated fluxes and the initial rough estimate of the fluxes is minimized, subject to equations 1 and 2. This problem of least squares with equalities and inequalities is solved using the *lsei* routine from the LIM package [14]. These final estimates of changes in metabolism during disease are then compared with independent measurements of the changes in metabolism during the disease state that had not been used in the estimation process. For additional explanation see Results. For convenience we term our new algorithm to predict changes in metabolic fluxes from changes in gene expression Least-squares with equalities and inequalities Flux Balance Analysis (Lsei-FBA).

## Results

### Example of a Flux Balance Analysis with BiGGR

To demonstrate the functionality of BiGGR, we conducted a flux balance analysis using a metabolic model that was previously used to study the effect of physical exercise on glucose and lactate metabolism in the human brain [10]. The small model represents the core of brain energy metabolism. We used the small model to estimate metabolic fluxes in the normal brain. Please note that all code necessary to reproduce the analysis shown here can be found in the package vignette of the BiGGR Bioconductor package.

The model consists of 89 metabolites and 71 reactions representing the glycolytic pathway, the pentose phosphate pathway (PPP), the citric acid cycle, malate-aspartate shuttle, the glutamate and GABA shunt and oxidative phosphorylation in the brain and was assembled from the Recon 1 reconstruction database. A list of full reaction equations, metabolites and a model scheme of the whole network is given in S1 and S2 Tables and the S1 Fig., respectively. Cerebral rates of metabolite uptake and release had been determined previously in five older (55–65 years) human subjects at rest [25] and the median values were used as equality constraints in the analysis. Assuming a brain mass of 1.4 kg for a healthy, adult human [26] we calculated uptake rates of 0.284±0.058 mmol/min for glucose (median±standard deviation), -0.013±0.032 mmol/min for lactate and-0.003±0.004 mmol/min for pyruvate. The standard deviations reported here were estimated from the ranges reported for the original data using an approach published by Hozo et al. [27]. Note that a negative uptake rate means that the brain releases the metabolite. Other measurements had shown a small flux in the he PPP in the normal brain, which amounts to 6.9% of glycolysis [28]. Flux through the GABA shunt accounts for 32% of the total glucose oxidation in the brain [29]. To account for these facts, fluxes of the glutamate decarboxylase producing GABA and the glucose-6-phosphate dehydrogenase were therefore set to 0.091 and 0.020 mmol/min, respectively, representing the appropriate fractions of glucose uptake.

With the constraints given above, we optimized the network for maximal ATP synthesis. To this end, the net balance of ATP producing and ATP consuming fluxes is maximized:

$$v_{ATPsyn}-\;v_{NDPK}-\;v_{Hex}-\;v_{PFK}-\;v_{PGK}+\;v_{PYK}$$ (4)

Where ATPsyn is the mitochondrial F0/F1 ATPase producing ATP from ADP and inorganic phosphate, NDPK the mitochondrial nucleoside-diphosphate kinase which usually carries a negative flux corresponding with ATP production. Hex (hexokinase) and PFK (phosphofructokinase) consume ATP to phosphorylate glucose. PGK (phosphoglycerate kinase) and PYK (pyruvate kinase) usually produce ATP (the PGK flux is negative in that case).

**Fig 2:**
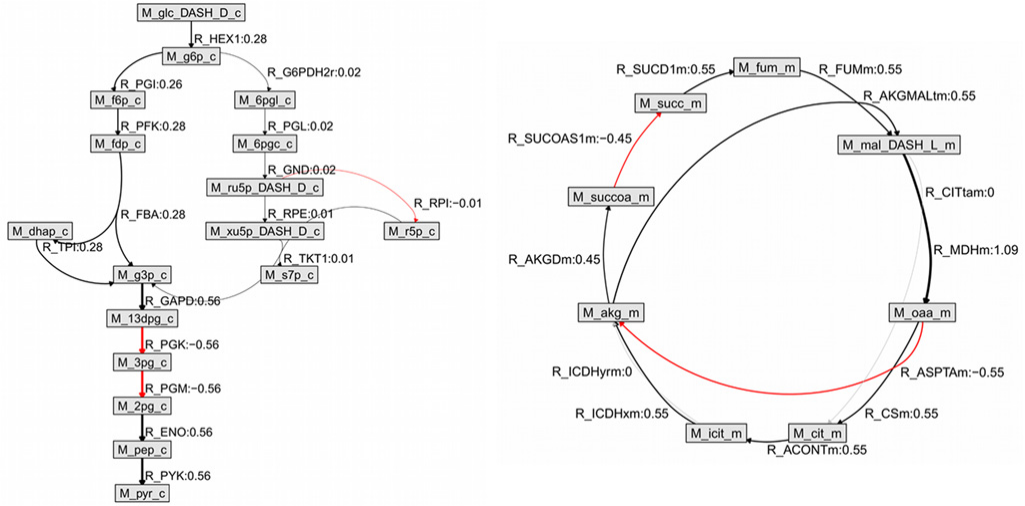
Automated plot of estimated fluxes in a model of brain energy metabolism.

The resulting maximal net ATP production (the objective function value) given the measured uptake of glucose and release of lactate and pyruvate was calculated to be 8.49 mmol/min with a mitochondrial ATP synthase flux of 7.48 mmol/min. Numerical values for all flux estimates are given in S3 Table. Flux Variability Analysis [30,31] showed that the flux distribution at maximal ATP synthesis provides a unique solution. To visualize the estimated fluxes, subsets of the metabolites in the model representing the glycolytic pathway and parts of the PPP (Fig. 2, left) and the citric acid cycle (Fig. 2, right), respectively, are automatically plotted using BiGGR.

Grey boxes represent metabolites, edges (arrows) represent reactions. Labels for boxes and edges show the Recon 1 identifiers for metabolites and reactions, respectively. Estimated flux values in mmol/min for the whole brain (rounded to two decimal places) are plotted together with the reaction identifier. Note that only part of the whole network is displayed. The left hand side of the plot shows the reactions in the glycolysis pathway and a subset of the reactions of the pentose phosphate pathway. The right-hand side shows the citric acid cycle. Edge widths are proportional to the estimated reaction fluxes. Arrows point in the direction of the calculated flux. Negative fluxes (reaction runs in reverse direction relative to the one defined as forward in Recon 1) are plotted in red and the flux values are printed as negative. R_AKGMAL is a transport reaction across the mitochondrial inner membrane exchanging alpha-ketoglutarate for malate. Note that not all metabolites involved in a reaction are always plotted. For instance, R_ASPTAm is a transamination reaction inside the mitochondrial matrix which involves aspartate and glutamate (not in scheme) in addition to M_oaa_m and M_akg_m which are plotted.

### Example of sampling feasible flux distributions using *in vivo* data

Experimentally determined exchange fluxes for metabolic systems are subject to measurement error. Internal fluxes in the system can be estimated by model analysis using the exchange fluxes as input. It is desirable to quantify the effect of measurement error on the estimates for the internal fluxes. The uncertainty of estimated parameters can be quantified by sampling an ensemble in parameter space which describes the experimental data within precision limits posed by the measurement error [21]. The resulting parameter ensemble then directly reflects the uncertainty in the estimated parameters based on the measurement error of the data given as input.

BiGGR provides the functionality to sample a posterior parameter ensemble using the Metropolis-Hastings algorithm in constrained linear systems, implemented in the *xsample* algorithm [18]. In the FBA described in the previous section, the values for uptake and release of various substrates, determined experimentally, were entered into the FBA procedure as equality constraints. In contrast, in this section we take possible variation of the *in vivo* flux measurements [25] into account in our flux estimation procedure: we entered the measured values and their standard deviations as approximate rather than exact equality constraints in the model (equation 3, see Methods section for details). In addition, fluxes for glucose-6-phosphate dehydrogenase and glutamate decarboxylase were constrained to be 6.9 and 32% of glucose uptake, respectively, exactly as in the FBA of the previous section.

We then sampled an ensemble consisting of 100000 parameter sets, allowing the measured fluxes to deviate according to normal probability distributions with the given standard deviations. As a starting point for the Markov chain, we used the parameter set resulting from the FBA performed above. It is important to note that all parameter sets still satisfy all other constraints posed by the model, such as the steady state and reaction irreversibility constraints (see Methods section for further details). Initial ensemble simulations showed that, given the uncertainty in the measured metabolite uptake and release rates, a relatively high flux in the glycerol-phosphate shuttle was predicted, which in turn lead to reversal of the malate-aspartate shuttle. In the ensemble simulation reported here we therefore additionally constrained the flux of the glycerol-3-phosphate dehydrogenase to zero. Mean values and standard deviations of all fluxes in the ensemble are given in S3 Table.

**Fig 3:**
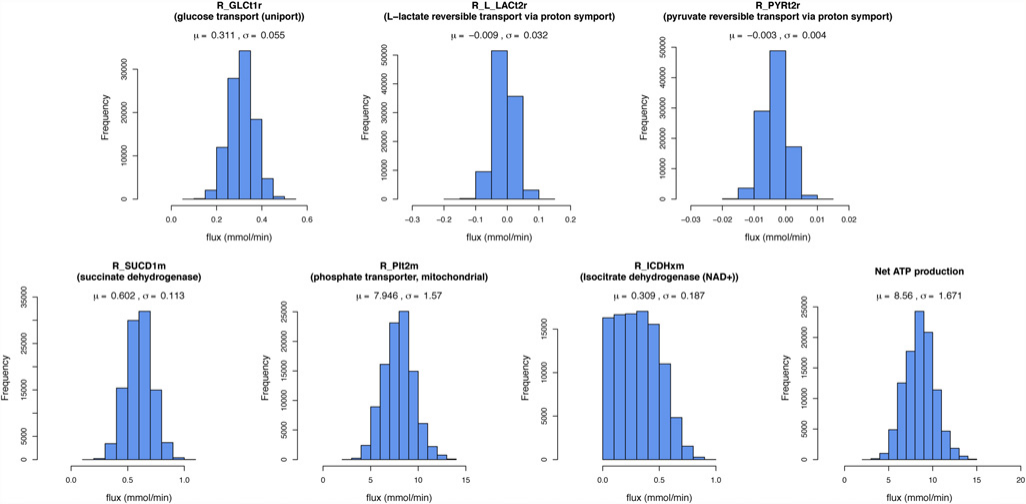
Ensemble analysis of uncertainty of flux estimations for brain metabolism.

Histograms for fluxes together with their mean and standard deviation within the ensemble are plotted in Fig. 3. Due to the assumption of metabolic steady state, the values of many fluxes in the ensembles are equal, for example the fluxes for phosphofructokinase and aldolase, since they are connected in series without branch points. We therefore only plot the histograms for a selection of all estimated fluxes.

Shown are histograms for selected fluxes of a flux parameter ensemble sampled with Metropolis-Hastings. The top row shows exchange fluxes of metabolites, the bottom row predicted internal fluxes. The spread of the histograms reflects the potential spread of the estimates, based on the input measurements with their errors. The estimates are subject to the constraints on the network. The size of the parameter ensemble was n = 100000. Mean and standard deviation for the flux values in the ensemble are given above each histogram.

With the ensemble sampling method, we can directly identify the uncertainty of the flux estimates given the error on the measured exchange fluxes. The upper four histograms in Fig. 3 show the ensemble results for these exchange fluxes while the bottom row shows estimates for internal fluxes.

Some estimated fluxes that had not been directly measured, e.g. for succinate dehydrogenase, show relatively well defined peaks and low standard deviations in the ensemble (see Fig. 3). In particular, the ensemble simulation suggests potential variability in the way malate is transported into the mitochondria as part of the malate-aspartate shuttle: while all malate flux into the mitochondria is via the alpha-ketoglutarate/malate transporter according to the FBA results, a substantial fraction of the malate transport goes via the citrate/malate exchange reaction in a major part of the ensemble (see S3 Table). This explains why the flux for isocitrate dehydrogenase also shows a larger spread in the ensemble. However, despite such variation in internal fluxes the net ATP production rate is predictable with relative precision (see Fig. 3). Note that the maximized ATP synthesis value resulting from the FBA in the previous section, 8.49 mmol/min, is within the range predicted by the ensemble method.

An uncertainty measure on the flux estimate represents valuable information on what is known on the functioning of the biological system encoded in the model. The sampling of posterior parameter ensembles to assess the uncertainty of the flux estimates and reveal potential alternative metabolic patterns has therefore significant advantages to complement an FBA procedure.

### Example of flux prediction during a disease from gene expression data

We illustrate in the following the integration of “omics” data such as gene expression using BiGGR jointly with other Bioconductor tools. The aim was to predict changes in fluxes. As an example we shall predict fluxes during a disease state from gene expression data, using mRNA expression measurements from Alzheimer’s disease (AD) patients against healthy controls [23] in the hippocampal region of the brain.

Available methods to predict changes in fluxes from changes in gene expression work best when there are large changes in gene expression, but their assumptions seem less applicable when there are many small, largely congruent changes in gene expression in a pathway (see Discussion). The latter is applicable to changes seen in the brain during neurodegenerative diseases. To tackle this situation, our method is based on the bold assumption that fluxes for each enzyme tend to change proportionally to the changes in mRNA expression. This assumption is likely not true on the level of a single enzyme, but applies under some conditions by reasonable approximation to a large metabolic network [32]. We assume that if many modest changes in gene expression in a metabolic pathway point in the same direction that may be associated with a congruent real change in flux in that pathway. The lack of hard data on control strengths of enzymes, kinetic parameters that are valid *in vivo* in the brain, translation of gene expression into enzyme activity, etc., not only preclude an exact calculation of the effect of gene expression changes, but also makes it difficult to prove our assumption. However, we will test whether our simple assumption works reasonably well here by comparing predictions on changes of metabolism during disease with available measurements on patients of actual changes in metabolic fluxes. Please note that the measurements of changed metabolic fluxes during disease are not used in any way for the model prediction. If the comparison between predicted and independently measured fluxes during disease is favorable, this would increase the confidence in predictions of those fluxes in parts of the metabolic network for which no measurement is available.

The fluxes for the normal adult human brain at rest were obtained from the FBA with ATP synthesis maximized (see above). The fold changes in mRNA gene expression are subsequently used to provide an initial rough estimate of the changes in the metabolic network. To this end for each reaction in the network the flux estimate for the healthy brain is multiplied by the fold change of the expression of genes linked to that reaction. This first estimate is then refined by using the balance and irreversibility of fluxes in the metabolic network as additional constraints. To this end we minimize the sum of the squared deviations between the final estimates of the fluxes and the first rough estimates.

**Fig 4:**
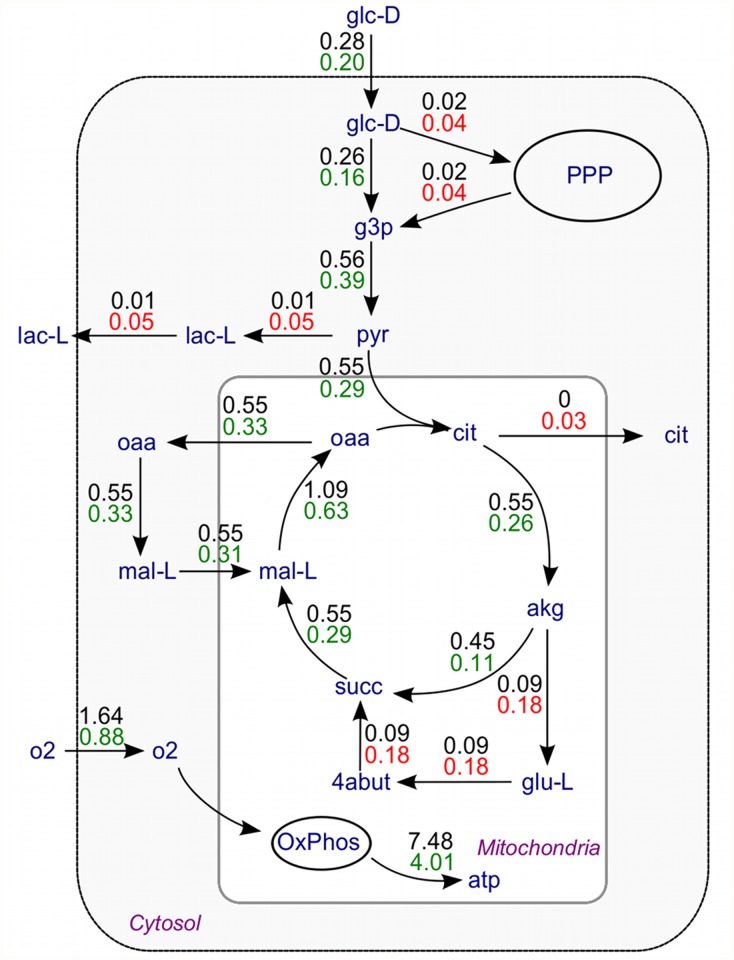
Flux distribution in a healthy brain and during AD.

Fig. 4 shows the flux distribution in the normal brain and during AD. During the disease, flux through glycolysis is predicted to be reduced by about 29% compared with healthy controls, while oxygen uptake into the brain is predicted to be reduced by 46%. Other pathways are also predicted to be reduced markedly during AD: the TCA cycle flux at the level of isocitrate dehydrogenase by 52%, mitochondrial ATP synthesis via oxidative phosphorylation by 46% and the alpha ketoglutarate dehydrogenase (AKGDm) reaction is reduced by 76%, which is compensated by the activation of the GABA shunt.

According to the prediction, the pentose phosphate pathway (PPP) increased twofold from normal during AD.

Predictions of flux distributions in normal healthy brain (top, black numbers) and during Alzheimer’s disease (bottom, red and green). Unit for flux is mmol/min for the whole brain. Fluxes in green represent decreases from the normal condition and red increases. Only major reaction fluxes are plotted to simplify network visualization. Metabolite names are according to the Recon1 reaction database. PPP: Pentose phosphate pathway, OxPhos: oxidative phosphorylation.

Our flux predictions agree with available experimental measurements. The decrease in metabolic rate in the temporal lobe measured by positron emission tomography (PET) was 77% for oxygen [33] and 36% for glucose [34]. Global cerebral metabolic rate for glucose (CMRglc) is reduced 20–25% in AD (reviewed in [35]). This compares with our calculated prediction of 46% and 29% reduction for oxygen and glucose, respectively. Thus, analysis from mRNA gene expression data leads to the prediction that energy metabolism in the brain is strongly compromised ([36]), particularly affecting reactions in the mitochondria. An estimated decrease by 50% in ATP production has been reported in sporadic AD [37], compared with 46% decrease in ATP synthase flux in our calculations. The model prediction is that lactate and pyruvate transport out of the brain is increased which agrees with increases in lactate and pyruvate levels measured in the cerebrospinal fluid of AD patients [38, 39]. Thus similar trends in metabolism in AD patients are seen in calculated predictions and independent measurements. In addition, the present analysis provides predictions for fluxes that have not been measured, which can among others be used for further assessment of the validity of the model if some of these fluxes are measurable in the future.

We also compared our predictions with the reported analysis of a larger multicellular model of brain metabolism [40]. In that study changes in fluxes were predicted based solely on a measured reduction in AKGDm activity rather than measured changes in gene expression for all reactions in the network as in our present analysis. The large model does not predict the decrease in overall ATP production predicted by our model and reported in [37] (see S4 Table for comparison of the predictions of both models). However, the large model predicts that AKGDm is bypassed via the GABA shunt, agreeing with our present prediction. This bypass helps to maintain aerobic metabolism.

## Discussion

The BiGGR open source package is built in R, is part of Bioconductor, and derives its input for model construction from metabolic reconstruction databases. BiGGR can be used to automatically construct mathematical and graphical representations of metabolic networks on the fly.

Many constraint based modeling software packages are currently available (see [7] for an overview), each having a different scope and flavor. Most of the tools are based on MATLAB, such as the COBRA Toolbox 2.0 [8], CellNetAnalyzer [41], FBA-SimVis [42] or the RAVEN toolbox [43]. Other tools are stand-alone software, for instance SurreyFBA [44] or OptFlux [45]. FAME is the first web-based approach to stoichiometric flux analysis [46].

BiGGR provides easy access to metabolic flux analysis for the large user base of the R environment. The vast number of open source analysis tools available in R is easily combined with the functionality in BiGGR. By supporting the modeling standard SBML and being embedded in the Bioconductor framework, BiGGR can be used in combination with other R packages, as e.g. sybil [11] or abcdeFBA [12]. A feature of BiGGR is the integration of metabolic reconstruction databases which facilitates model generation by querying the databases. Further, BiGGR directly supports Markov chain Monte Carlo methods to sample within the constrained solution space.

### Core model of human brain metabolism

Here we demonstrated the functionality of BiGGR by presenting a flux analysis of human brain metabolism. A model comprising glycolysis, PPP, citric acid cycle, oxidative phosphorylation and GABA shunt was automatically assembled within BiGGR from a list of reaction identifiers. We use a core model that represents all important ATP production processes including the glycolytic chain and oxidative phosphorylation. Recent mechanistic models of ATP metabolism in brain based on kinetic equations are of similar size and tend to contain the same reactions as included in our model [47,48]

This small model of the core of human brain energy metabolism was compared with a large model of human brain metabolism containing more than a thousand reactions and several brain cell types [40]. This comparison allowed to systematically examine branch points in the metabolic network connected with reactions that were neglected in the small model. From the comparison we concluded that the reactions quantitatively relevant for ATP production were adequately represented by our present small model. A detailed description and comparison of the differences between the large model of Lewis et al. and our smaller model is given in the S1 Text (‘*Comparison with a large*, *multicellular model of brain metabolism*’) and S4 and S5 Tables.

Tests showed that the calculation with our algorithm is feasible on models with the size of the Lewis model (>1000 reactions) [40] within fifteen sec of CPU time on an ordinary laptop. However, we have no gene expression data that are differentiated for the different cell types in the Lewis model and therefore do not report results for this large model.

As an example of applying BiGGR, we performed an FBA procedure with the objective to maximize net energy production of the brain cell with the small model. Published measurements of cerebral uptake and release rate of metabolites in the brain of healthy elderly control subjects ([25]) were used as input to the analysis. Parts of the model and estimated fluxes were visualized using BiGGR (Fig. 2). An MCMC procedure was then used to assess the uncertainty of the flux estimates. To this end, an ensemble of possible flux configurations that agree with the measurements of cerebral metabolic rates was generated (Fig. 3). We then used the model to predict how fluxes are changed during Alzheimer’s disease compared to healthy subjects (Fig. 4). For this analysis, fold changes in gene expression measured by microarray experiments were taken into account. Model predictions show the same trends as PET measurements of cerebral metabolism during Alzheimer’s disease (see Results). An extensive analysis of the prediction of flux changes based on gene expression changes in other neurodegenerative diseases and other brain regions will be published separately.

### Objective function for analysis of brain metabolism

An appreciable part of the total energy turnover of the human body at rest takes place in the brain. It seems reasonable to assume that, given a certain uptake of nutrients to support energy metabolism, ATP synthesis is maximized. Several alternative objective functions for models of metabolism in brain have been discussed by Cakir et al. [49] (see their S2 Table). Maximal ATP production was one of the objective functions considered. The drawbacks of maximal ATP production are considered by Cakir to be the predicted inactivity of the pentose-phosphate pathway flux and zero flux in the GABA cycle. Note that these disadvantages of maximal ATP production as objective function were avoided in our approach by constraining the PPP and GABA shunt fluxes. The third deficit mentioned by Cakir et al., low lactate release flux, is actually in good correspondence with the measurements in the control group of elderly healthy people which we used. Cakir et al. considered maximization of the glutamine/glutamate/GABA cycle a reasonable objective function for brain metabolism. This cycle is not a focus of our present model which does not include multiple cell types because we aim to analyze gene expression measurements made on whole brain tissue and not differentiated to different cell types. However, note that the glutamine/glutamate cycle in Cakir’s model is a ‘futile cycle’ from the biochemical point of view which requires ATP splitting. Because this cycle is very active, forming a major part of total ATP hydrolysis, maximizing ATP turnover is compatible with a very active glutamine/glutamate cycle. Given that our model focuses on ATP producing processes, maximizing ATP production seems a reasonable objective.

The adult brain is not growing, and therefore the emphasis on biomass components as seen in objective functions for metabolic models of microorganisms is not appropriate. However, to produce a reasonable baseline flux distribution, not only ATP synthesis was considered, but other metabolic functions in the brain were taken into account: the pentose phosphate pathway flux and the GABA shunt flux were set to fractions of glucose uptake that are representative of measurements in normal brain cells. We therefore consider ATP the limiting commodity given a certain level of measured nutrient uptake, while considering additional constraints to take other aspects of brain metabolism into account.

### Predicting metabolism from gene expression

Here we introduce an algorithm termed Lsei-FBA to predict changes in metabolic fluxes from measured changes in gene expression. Several algorithms to predict metabolism from gene expression changes have been designed previously [50]. Some of these algorithms use coarse, discrete levels of gene expression, which is less suited for data from diseased tissue which often shows relatively modest changes in gene expression. Many of these algorithms are not suited for the situation where measurements of metabolite exchange during disease are not available. We discuss some of the best known algorithms here.

The iMAT algorithm [51] predicts which biochemical reactions are active in a certain organism. It subdivides gene expression levels in low, normal and high. Note that quantitative prediction of fluxes from modest, continuous changes in gene expression is not a suitable application area for iMAT. The GIMME method [52] has the same application area as iMAT, but minimizes a linear combination of fluxes whose associated gene expression is below a certain threshold, with weights for the fluxes to be optimized proportional with the degree of gene expression below the threshold value. At the same time metabolic functions are maintained at relatively high minimum values. MADE [53] sets expression levels to 0 or 1 without invoking an explicit threshold, based on the statistical significance of changes in expression levels. Setting gene expression to merely two discrete levels is much too coarse for our application where gene expression levels change modestly and in a continuous fashion. Lee et al. [32] replace the usual cellular objective function (maximal growth or ATP production) with an objective function based on the correspondence between absolute gene expression and estimated flux. Although this yields good results to predict metabolic flux in an organism, especially if the measurement yields an absolute measure of gene expression, this approach does not directly apply to predictions based on changes in gene expression between two conditions. E-Flux [54] models maximum flux constraints based on measured gene expression.

The GX-FBA algorithm was developed by Navid and Almaas [55] and applied to gene expression data of the bacterium *Yersinia pestis*. In their algorithm use is made of nutritional constraints specific for the test condition, which is not directly compatible with our application area. Reversible reactions are not taken into account in GX-FBA. The upper and lower bounds of reactions are modified, and the upper bound for a reaction may for instance be set lower than original even if gene expression for that reaction is increased. Their cost function maximizes fluxes for which gene expression is upregulated and minimizes fluxes for which gene expression is down regulated, weighted by the logarithm of the ratio of gene expression between test and control. Note that although the weight of a flux in the cost function increases with the logarithm of the fold change of gene expression, the flux is not optimized to be near the fold change times the wild type value as in our algorithm.

Machado and Herrgard [50] compared the performance of seven of such algorithms used to make predictions about metabolic systems based on gene expression measurements on yeast and E. coli. We ran the algorithms in the Machado framework on our brain model and data, making use of the code provided by Machado. The results of several algorithms are summarized in S6 Table. However, the test condition for these algorithms was necessarily entirely different than for the case of these microorganisms, because Machado and Herrgard made use of the measured uptake of nutrients not only for the control condition but also for the test conditions. The uptake of glucose and oxygen measured separately under each test condition in yeast was for instance given as input to the algorithms, in addition to gene expression. This is not compatible with our application area where there are uptake measurements for the control condition, but the uptake under disease conditions has not been measured and must be estimated using our algorithm.

A further important difference is that Machado tested the algorithms on bacteria and yeast where in several of the algorithms the growth rate was maximized. The results of Machado show that the predictions made by the algorithms for the fluxes in the microorganisms were of limited quality. It is therefore remarkable that the results of our algorithm on brain disease were reasonable.

Our application area is to estimate changes in fluxes, including uptake rates, in the disease condition without the availability of measurements of uptake rates during disease. Because the exchange rates during disease have not been measured, we tested two extreme situations: (1) the exchange rates are given as measured in the control condition or (2) the upper bound for the exchange rate was set to an unrestrictive value (2 mmol/min). For test 1 GIMME gives the same result as flux balance analysis under the control condition, despite that the FBA for the control does not take the gene expression for the disease data into account, as opposed to GIMME. For test 2 GIMME gives fluxes and uptake rates that approach the maximum values compatible with the upper bounds imposed in the model, e.g. 2 mmol/min for glucose uptake, which is equal to the imposed upper bound. Given the nature of the GIMME algorithm which tends to maintain metabolic functionality and given the modest changes in gene expression in the AD data set, these results were to be expected.

We also tried the implementation in the Machado test suite of the Lee-12 algorithm [32], which is based on the assumption that maximal correspondence between gene expression and fluxes provides a good objective function for predictions. This algorithm was shown to outperform classic objective functions for yeast. Run in the Machado test suite on our model a deviant pattern of metabolism was found: for instance, pyruvate uptake was predicted to be higher than the measured glucose uptake and lactate release was four times the glucose uptake in Alzheimer patients (see S6 Table). The predictions of Lee-12 for Alzheimer patients were in sharp contrast to measurements of Lying-Tunell et al. in suspected Alzheimer disease [25].

Similar observations were made for the E-Flux algorithm which also predicts a very deviant pattern of metabolism. High lactate and pyruvate release was predicted in controls and AD patients [25]. The results in S6 Table demonstrate that most of the algorithms tested by Machado perform less well than our new Lsei-FBA algorithm for the condition that exchange fluxes of metabolites between tissue and blood are not known. However, it is possible that these other algorithms are useful if metabolite exchange has been measured and can be used as a constraint.

### Mapping genes to biochemical reactions

When multiple genes are associated with a biochemical reaction, we average the fold changes of gene expression. This is perhaps the simplest possible choice to map gene expression to the reactions. One finds a range of approaches in the literature. Lewis et al [40], for instance, also averaged gene expression in an analysis with their model of brain metabolism. Fang et al. [56] took the geometric mean of the expression of all genes associated with a reaction, Navid and Almaas [55] take the value of the gene that deviated most from 1 (either up or down) and when genes associated with a certain reaction are inconsistent in their direction of up- or downregulation, the reaction is not taken into account. The approach by Lee et al. [32], taking the minimum of gene expression for all genes associated with an enzyme complex, seems plausible, although perhaps a complex which lacks a certain peptide can still be partially active. One can even imagine an inhibitory subunit being downregulated in a complex, leading to increased enzyme activity. The choice for the sum of the components when alternative enzymes (isoforms) are involved may be regarded as equivalent to taking the average of gene expression changes as done in the present analysis. Machado and Herrgard [50] in their comparison of several algorithms seem to use the different approaches taken in the original papers on the tested algorithms. To the best of our knowledge, hard empirical evidence supporting one choice or the other to map gene expression to a reaction has not yet been reported. We chose to apply perhaps the simplest possible assumption, averaging of the expression of genes associated with a reaction, in our present example calculation. However, algorithms for more complex mappings of gene expression to the associated reactions have been added to BiGGR.

### Testing model predictions with independent data

The model contains more than seventy fluxes and the flux distribution is estimated based on six measured fluxes (four exchange fluxes and two flux ratios for the pentose phosphate pathway and GABA shunt respectively). The exchange data can of course be precisely fitted by the model. This does not add further proof to the correctness of the model which is based on the extensive biochemical literature, but instead the experimental data are used to calibrate the fluxes in the control condition. Using Flux Variability Analysis we showed that the solution for the internal flux distribution in the control condition is unique.

To predict the metabolic flux pattern in the patient group we make use of two distinct sources of data: (1) the flux distribution for the normal brain as described above, and (2) gene expression measurements from Alzheimer’s disease (AD) patients in comparison with a control group. We predict the metabolic fluxes in AD patients based on data source 1 and 2, using the Lsei-FBA algorithm. The final predictions of changes of metabolic fluxes during AD from the control condition are then compared with data source 3: independent in vivo measurements of metabolic changes (glucose and oxygen uptake, ATP synthesis, lactate and pyruvate levels) in AD patients compared to controls (data source 3). Note that the data from source 3 were not fitted by the model, but used to test the model by comparing with the model predictions.

### Determining variability of estimates by sampling

The procedure of sampling posterior ensembles of possible flux distributions that agree with measured input fluxes is based on Bayesian parameter inference. Knowledge of the measurement error of known fluxes is thereby used as prior information. Compared to an FBA analysis based on cost or profit function optimization, sampling a distribution of possible flux vectors has the advantage that uncertainty in the estimated fluxes can be directly quantified. As a result, confidence bounds can be defined on flux estimates that are difficult to measure directly. The method thus allows for assessing the robustness of estimates based on a metabolic model. The incorporation of prior knowledge on measured exchange fluxes helps to better delimit feasible and infeasible regions in flux space. Sampling the multidimensional flux space based on hypothetical exchange fluxes with very permissive upper and lower bounds without providing accurately measured prior values could still be used, e.g. to identify whether reactions are used at all or are correlated. Another approach to assess the range of possible fluxes in constraint based models is Flux Variability Analysis (FVA) [30,31]. FVA differs from our approach because it quantifies the possible variation in each estimated flux separately, e.g. to assess which values for a flux are compatible with the unique optimal value of the objective function. Also for FVA setting realistic upper and lower bounds on blood-to-tissue exchange fluxes based on measurements may be useful to delimit feasible flux ranges more precisely. However, in contrast to the ensemble approach, FVA does not reveal the correlation of different reactions.

A Bayesian approach to FBA was applied by Heino et al [57]. Posterior flux distributions for a model of skeletal muscle metabolism were estimated using MCMC sampling. Other than in the ensemble sampling approach described here, the method in [57] takes the objective function to maximize ATP production into account as prior information in the model analysis and also bound constraints were used as priors. The MATLAB-based software METABOLICA developed by the same group [58] also allows for setting Gaussian prior probabilities, similar to the ensemble sampling presented here. We chose to use Gaussian priors on measured input fluxes in our analysis to best reflect the measurement uncertainty in the estimated posterior distribution. However, prior probabilities on bounds and on objective function values could be easily added due to the modular design of BiGGR.

## Conclusions

The Bioconductor R package BiGGR facilitates constraint based modeling using metabolic reconstruction databases. Special emphasis was placed on the functionality to query metabolic reconstruction databases for pathways, reactions, metabolites or genes to compile constraint-based metabolic models. Metabolic fluxes can be estimated by interfacing linear programming routines. Further, metabolic models and estimated fluxes can be automatically visualized and integration with Markov chain Monte Carlo methods allows for sampling of possible flux configurations. BiGGR is open-source, free of charge and platform independent. As examples of the application of BiGGR, we predicted the metabolic flux distribution in the normal human brain and derived predictions of changes in metabolic patterns during Alzheimer’s disease using the new Lsei-FBA algorithm based on gene expression measurements.

## Supporting Information

S1 Fig Reconstructed metabolic reaction network for brain metabolism. (TIF)

S1 Table List of reactions included in this model after extraction from the Recon1 knowledgebase for human metabolism. (XLSX)

S2 Table List of metabolites included in this model, extracted from the Recon 1 knowledgebase. (XLSX)

S3 Table Flux distribution in normal humans and during AD. (XLSX)

S4 Table Flux distributions for all reactions calculated using the FBA method and estimation of uncertainty analysis. (XLSX)

S5 Table Common metabolite comparison between Lewis and present model. (XLSX)

S6 Table Result tests of algorithms in Machado framework on brain model. (XLSX)

S1 Text Comparison with a large, multicellular model of brain metabolism. (DOC)
