# Supplementary figures and images for: Using Bioconductor Package BiGGR for Metabolic Flux Estimation Based on Gene Expression Changes in Brain
Source: PLoS One. 2015 Mar 25;10(3):e0119016. doi: 10.1371/journal.pone.0119016 (PMC4373785; doi:10.1371/journal.pone.0119016)

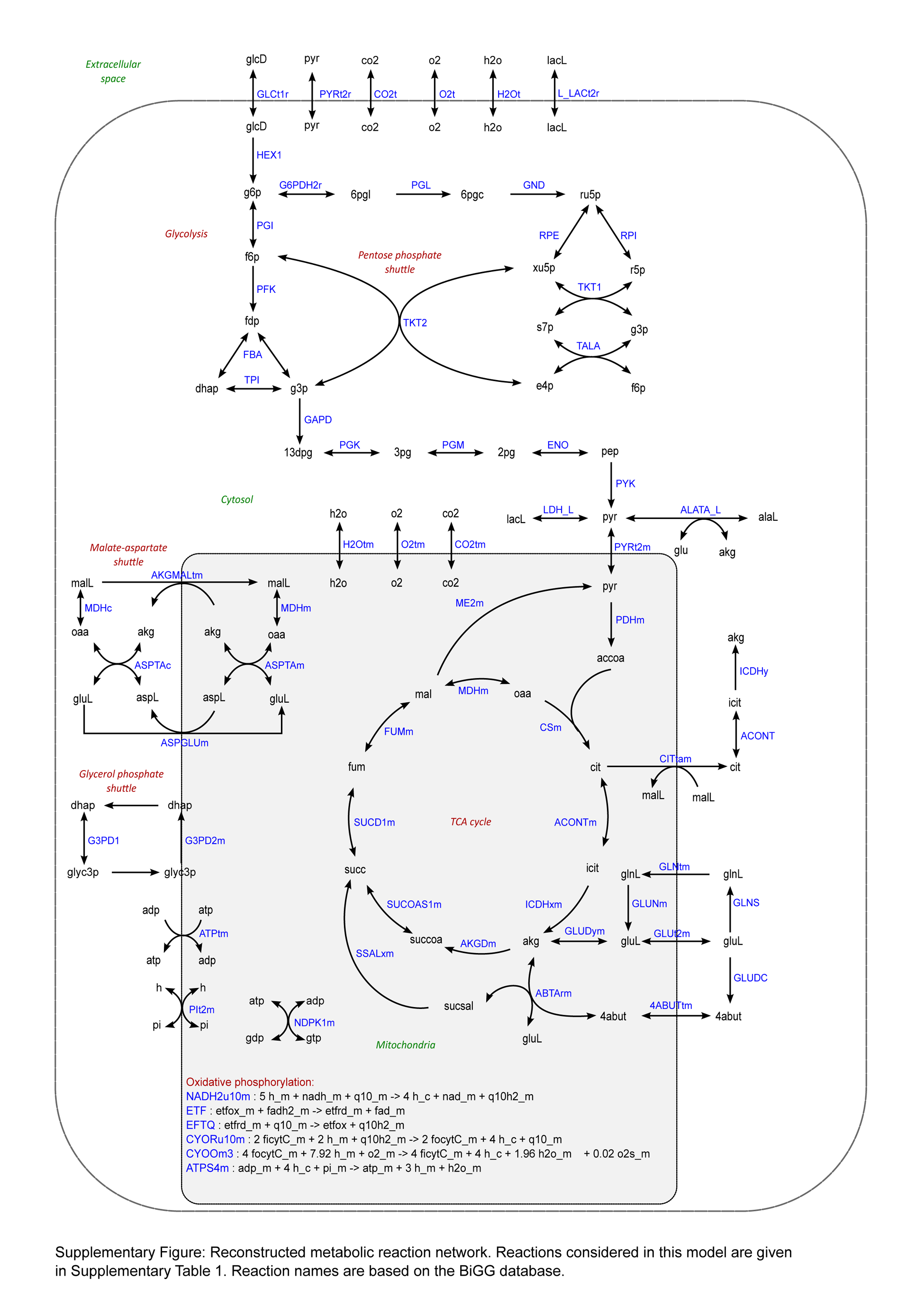

Supplement: S1 Fig — (TIF) [file pone.0119016.s001.tif]
