# Supplementary material for: Using Bioconductor Package BiGGR for Metabolic Flux Estimation Based on Gene Expression Changes in Brain
Source: PLoS One. 2015 Mar 25;10(3):e0119016. doi: 10.1371/journal.pone.0119016 (PMC4373785; doi:10.1371/journal.pone.0119016)
Supplement: S1 Text — (DOC) [file pone.0119016.s008.doc]

Supplementary notes:

***Comparison with a large, multicellular model of brain metabolism***

A number of energy metabolism models for the brain have already been developed that include interaction between astrocytes and neurons. Some of these models have been compared in the supplementary material in the article of Lewis et al. (2010) [1]. Of these models, the model of Lewis et al. is the largest and most detailed. Here, we compare our model with the Lewis model.

***Metabolic network reconstructions***

The Lewis model contains a total of 1073 reactions comprising energy metabolism and general metabolic reactions modeled for three neuronal cell types: glutamatergic, GABAergic and cholinergic. In contrast, our model was developed focusing on ATP synthesis, incorporating reactions from Recon1. Our model was curated by examining the biochemical literature and the models of Cakir et al. [2] and Occhipinti et al. [3] with the aim to capture the reactions that are important for ATP synthesis. Our model overlaps strongly with the Lewis model which also derives from Recon1. Our small model contains 71 reactions and is combined with an objective function to maximize ATP production in the brain while constraining some other pathways (pentose phosphate pathway, GABA shunt) to be a specific fraction of glucose uptake.

Two reactions used in our model are different from the analogous ones in the Lewis model. These reactions are highlighted in Supplementary Table 4 of this manuscript. Our model includes the generation of superoxide in the cytochrome oxidase reaction in contrast to the model by Lewis. The Lewis model considers both ADP and GDP forming succinate CoA ligase in the initial model, although in the reduced model used by Lewis for ensemble generation [1] only the ADP forming reaction is considered. Our model contains the GDP forming succinate CoA ligase, but the GTP formed during the normal operation of this enzyme in the TCA cycle immediately forms ATP in the NDPK1 reaction, making the overall result the same. The Lewis model considers both sodium and non-sodium coupled transport reactions in the initial model, but in the reduced model, only non-sodium coupled reactions are considered.

***Differences in estimated exchange fluxes***

The input parameters for the exchange reactions were taken from Lying-Tunnel et al. [4]. Lewis et al. use the data of young subjects as reference while we use data from the elderly subjects because we consider this better suited as a control group for Alzheimer’s disease patients.

The results of Lewis contain some notable thermodynamically infeasible loops, i.e. loops of fluxes which are mathematically feasible but form internal loops in the model. Infeasible loops can be seen in some of the reactions in Supplementary Tables 4 and 5, often consisting of very large fluxes that are connected in the thermodynamically infeasible loops (e.g. see R_NDPK1m and R_GLNtN1 in Supplementary Table 4A). A method to remove these loop reactions in the network has been designed by authors of the Lewis model [5].

In Supplementary Table 5 an overview is presented of all metabolite nodes present in our present model, which are all also included in the Lewis model. In this Table flux values of reactions present in both models can be compared with results for reactions only present in the Lewis model. This comparison suggests that removing reactions from the large model resulting in a small well-curated model was justified.

***Glycolysis to pyruvate***

During glycolysis, transfer of phosphate group from 1,3-diphosphoglycerate (13dpg) to 3-phosphoglycerate (3pg) is commonly thought to occur via the ATP generating reaction phosphoglycerate kinase (PGK), as included in our model. This is a preferred pathway to maintain efficient ATP synthesis by the glycolytic flux. In the Lewis model, two other alternative routes exist, via acylphosphatase (ACYP), or via diphosphoglycerate mutase (DPGM) and diphosphoglycerate phosphatase (DPGase). Only a fraction of the fluxes goes via PGK in the Lewis model (30%, 11% and 14% in glutamatergic, GABAergic and cholinergic cells, respectively). In each of the two other alternative pathways, one molecule of phosphate is hydrolyzed and no net ATP is produced.

ACYP is present in many multicellular organisms including humans. However, the physiological properties of this enzyme remain largely unknown. Evidence for ACYP as a reaction that may bypass PGK in the cytosol is reviewed by Stefani et al. [6]. However, in post mortem brain of Alzheimer’s disease patients, no changes in the activity of this enzyme are detected [7].

Similarly, the conversion of 13dpg to 3pg via the bifunctional enzymes DPGm and DPGAse (known as the BPG shunt) may not be a preferred “bypass” reaction for the brain, since no net ATP is generated. The intermediate of this pathway, 2,3-diphosphoglycerate (23dpg), is known to be found uniquely in erythrocytes and placenta [8].

Furthermore, there is a small flux from 3pg to 3-phosphohydroxypyruvate (3php), an intermediate metabolite towards synthesis of serine, via astrocytic enzyme phosphoglycerate dehydrogenase (PGCD), which carries 12.75%, 10.72% and 12.69% of the flux from the 3pg pool for glutamatergic, GABAergic and cholinergic cells, respectively in the Lewis model analysis.

The neuronal enzyme phosphoenolpyruvate carboxykinase (PEPCK) contributes about 10%, 0.4% and 15% of fluxes in glutamatergic, GABAergic and cholinergic cells, respectively, towards production of phosphoenolpyruvate (pep).

In the citric acid cycle, a large flux is observed in the Lewis model through the reaction of NAD dependent isocitrate dehydrogenase (ICDHxm), which is about twice the flux from the preceding reaction aconitase (ACONTm) in all three cell types. This large flux is compensated by the backflux via the NADP dependent version of this reaction (ICDHym). Subsequently, the conversion production of succinyl-CoA to succinate goes via two enzymes in the Lewis model: ADP-forming succinate-CoA ligase (SUCOASm), but also via 3-oxoacid CoA-transferase (OCOAT1m) which often carries an appreciable flux in the Lewis model but is not present in our model.

Fluxes that utilize ATP or GTP in the Lewis model at appreciable rates but are missing in our model:

mitochondrial nucleotide synthesis and mtDNA repair

C5 – branched dibasic acid metabolism

folate metabolism

vitamin D metabolism

itaconate and mesaconate metabolism

creatine/phosphocreatine shuttle

These pathways, which consume rather than produce ATP are beyond the scope of our present model, which focuses on ATP production and lumps ATP consuming processes.

***Comparison with experimentally measured data***

Values are in mmol min^-1^ for the whole brain with the assumption that the brain weight is 1400g (Lewis et al., 2010). Lewis et al. flux data are the average of the sampling points.

|  |  | Lewis et al., 2010 | | | This study |
| --- | --- | --- | --- | --- | --- |
|  | Literature values | GABAergic | Cholinergic | Glutamatergic |  |
| CMRO2 | 2.464 [2], 2.1 [9], 2.464 (young subjects) [4], 2.3506 (old subjects) [4] | 3.0464 | 3.1052 | 3.0870 | 1.6386 |
| CMRglc | 0.448 [2], 0.322-0.42 [9], 0.3472 (young subjects) [4], 0.2842 (old subjects) [4] | 0.4041 | 0.3949 | 0.3977 | 0.2842 |
| CMRCO2 | 2.3912 -2.464 [2] | 2.8530 | 2.5270 | 2.5396 | 1.6565 |
| CMRO2/CMRglc | 5.5 [2], 5 [9] | 7.53 | 7.86 | 7.76 | 5.76 |
| CO2/O2 (Respiratory quotient) | 0.97 [9] | 0.93 | 0.81 | 0.82 | 1.01 |

***Lactate metabolism***

There has been some debate on the existence of an intercellular lactate shuttle. This hypothesized form of intercellular transport is termed the astrocyte-neuron lactate shuttle (ANLS), in which lactate is transported from astrocytes to neurons as an energy source (for review, see [10]). In contrast to this hypothesis, in the Lewis model analysis, lactate in shuttled from neurons to astrocytes. Our small model does not include different cell types and lactate transport between the cells is not considered.

According to the intracellular lactate shuttle (ILS) hypothesis, lactate metabolism in the cytosol via glycolysis/gluconeogenesis is balanced by lactate oxidation in the mitochondria [11]. In the Lewis model cytosolic lactate is transferred into the mitochondria and oxidized to pyruvate via a mitochondrial lactate dehydrogenase. This affects transport of reducing equivalents into the mitochondria (see below). Transport of reducing equivalents derived from NADH is therefore less via the malate-aspartate shuttle (AKG-MAL transport is low). Instead, NADH is produced more in the mitochondria via mitochondrial lactate dehydrogenase (LDH_Lm). Isocitrate dehydrogenase (ICDHxm) produces NADH at twice the flux from the precursor metabolite isocitrate. However, there is a backflux of isocitrate dehydrogenase which uses NADPH (ICDHyrm) which balances the total production of isocitrate (icit) and alpha ketoglutarate (akg). Evidence has been presented contradicting the intracellular lactate shuttle [12]. For the moment, we did not include the mitochondrial lactate dehydrogenase in the small model.

***Transport of reducing equivalents across the mitochondrial membrane via glycerol-3-phosphate dehydrogenase***

The malate-aspartate (MA) shuttle and the glycerol 3-phosphate (G3P) shuttle are two of the shuttles that transfer reducing equivalents across the mitochondrial membrane which is impermeable to NAD and NADH. The former shuttle is regarded as the most important shuttle in the brain [13].

The G3P shuttle is not as active as the MA shuttle. Unlike the MA shuttle, the G3P shuttle is irreversible and electrons are passed to FAD in the mitochondria instead of NAD, resulting in less ATP production [13]. The importance of G3P shuttle in the brain has been shown to be small [14], and metabolism of glucose and lactate was unchanged in the absence of MA shuttle. Nguyen et al. [15] regarded the G3P shuttle of little importance in the brain, based on the absence of one of the enzymes in the neurons, the cytosolic glycerol phosphate dehydrogenase. To complete the shuttle, both the mitochondrial and cytosolic version of the enzymes must be present in the same cell.

Regardless, it is worthwhile to include this shuttle in the model. It is not very active during FBA with ATP production maximized and appears to be removed from the reduced model of Lewis et al. (2010) but becomes quite active when the MA shuttle is being compromised. For this reason it is included in our small model.

References:

1. Lewis NE, Schramm G, Bordbar A, Schellenberger J, Andersen MP, et al. (2010) Large-scale in silico modeling of metabolic interactions between cell types in the human brain. Nat Biotechnol 28: 1279–1285. doi:10.1038/nbt.1711.

2. Cakir T, Alsan S, Saybaşili H, Akin A, Ulgen KO (2007) Reconstruction and flux analysis of coupling between metabolic pathways of astrocytes and neurons: application to cerebral hypoxia. Theor Biol Med Model 4: 48. doi:10.1186/1742-4682-4-48.

3. Occhipinti R, Puchowicz M a, LaManna JC, Somersalo E, Calvetti D (2007) Statistical analysis of metabolic pathways of brain metabolism at steady state. Ann Biomed Eng 35: 886–902. doi:10.1007/s10439-007-9270-5.

4. Lying-Tunell U, Lindblad BS, Malmlund HO, Persson B (1980) Cerebral blood flow and metabolic rate of oxygen, glucose, lactate, pyruvate, ketone bodies and amino acids. Acta Neurol Scand 62: 265–275.

5. Schellenberger J, Lewis NE, Palsson B (2011) Elimination of thermodynamically infeasible loops in steady-state metabolic models. Biophys J 100: 544–553. doi:10.1016/j.bpj.2010.12.3707.

6. Stefani M, Taddei N, Ramponi G (1997) Insights into acylphosphatase structure and catalytic mechanism. Cell Mol Life Sci 53: 141–151. doi:10.1007/PL00000585.

7. Liguri G, Taddei N, Nassi P, Latorraca S, Nediani C, et al. (1990) Changes in Na+,K(+)-ATPase, Ca2(+)-ATPase and some soluble enzymes related to energy metabolism in brains of patients with Alzheimer’s disease. Neurosci Lett 112: 338–342. doi:10.1016/0304-3940(90)90227-Z.

8. Pritlove DC, Gu M, Boyd CAR, Randeva HS, Vatish M (2006) Novel Placental Expression of 2,3-Bisphosphoglycerate Mutase. Placenta 27: 924–927. doi:10.1016/j.placenta.2005.08.010.

9. Clarke D, Sokoloff L (1999) Circulation and Energy Metabolism of the Brain. In: Siegel G, Agranoff B, Albers R, Mohnoff P, Fisher S, et al., editors. Basic Neurochemistry: Molecular, Cellular and Medical Aspects. New York: Raven Press. pp. 637–669.

10. Pellerin L, Magistretti PJ (2012) Sweet sixteen for ANLS. J Cereb Blood Flow Metab 32: 1152–1166.

11. Hashimoto T, Brooks GA (2008) Mitochondrial lactate oxidation complex and an adaptive role for lactate production. Med Sci Sports Exerc 40: 486–494. doi:10.1249/MSS.0b013e31815fcb04.

12. Sahlin K, Fernström M, Svensson M, Tonkonogi M (2002) No evidence of an intracellular lactate shuttle in rat skeletal muscle. J Physiol 541: 569–574. doi:10.1113/jphysiol.2002.016683.

13. McKenna MC, Waagepetersen HS, Schousboe A, Sonnewald U (2006) Neuronal and astrocytic shuttle mechanisms for cytosolic-mitochondrial transfer of reducing equivalents: current evidence and pharmacological tools. Biochem Pharmacol 71: 399–407.

14. Waagepetersen HS, Qu H, Schousboe a, Sonnewald U (2001) Elucidation of the quantitative significance of pyruvate carboxylation in cultured cerebellar neurons and astrocytes. J Neurosci Res 66: 763–770.

15. Nguyen NHT, Bråthe A, Hassel B (2003) Neuronal uptake and metabolism of glycerol and the neuronal expression of mitochondrial glycerol-3-phosphate dehydrogenase. J Neurochem 85: 831–842.
